# Short Imagery Rescripting Intervention to Treat Emotionally Dysregulated Behavior in Borderline Personality Disorder: An Exploratory Study

**DOI:** 10.3389/fpsyt.2020.00425

**Published:** 2020-05-20

**Authors:** Caroline Schaitz, Julia Kroener, Anna Maier, Bernhard J. Connemann, Zrinka Sosic-Vasic

**Affiliations:** Department for Psychiatry and Psychotherapy, University Clinic of Ulm, Ulm, Germany

**Keywords:** borderline personality disorder, imagery rescripting, dysregulated behaviors, emotion regulation, self-harm, mental imagery

## Abstract

**Background:**

Current research on borderline personality disorder report an association between emotionally dysregulated behaviors and intrusive mental imagery depicting similar scenes. Imagery rescripting techniques have proven effective in reducing intrusive mental imagery in numerous contexts. We developed a two session-short intervention in which intrusive mental images are identified, analyzed, and modified for daily rehearsal at home. This study aimed to reduce the negative emotions and cognitions associated with self-injurious behaviors by replacing unhealthy imagery with more adaptive content.

**Methods:**

Seven females diagnosed with borderline personality disorder who reported intrusive mental imagery of dysregulated behaviors were recruited for participation. Each participant engaged in two individualized treatment sessions and daily homework requiring the rehearsal of modified imagery. Emotion regulation strategies, borderline symptom severity, and depressiveness were assessed before and after treatment.

**Results:**

Acceptance was positive, as no patient dropped out from treatment. Symptom exacerbation was not observed. Borderline symptom reduction was noted and indicia of emotional dysregulation and negative affect declined.

**Limitations:**

The generalizability of results is limited by the small sample size and the absence of a control group. Conclusions: This new two-session short intervention was shown to decrease the emotionally dysregulated behaviors that accompany negative feelings in females with borderline personality disorder.

## Introduction

Up to 80% of individuals with borderline personality disorder (BPD) engage in self-injurious behavior without suicidal intentions. Various forms of impulsive, emotionally dysregulated behaviors (EDB) such as binge-eating, alcohol and drug abuse, and other high risk behaviors are frequently reported (1).

The first manualized, evidence-based psychotherapeutic treatment available for chronically suicidal patients was dialectical behavioral therapy [DBT; (2)]. Efficacy has also been reported for transference focused psychotherapy [TFP; (3)], mindfulness based therapy [MBT; (4)], and schema focused therapy [ST; (5, 6)]. These approaches may effect a diminution in symptoms, but usually last up to a 2 years treatment period. Drop-out rates range from 10% for ST (7, 8), 25% for DBT (9) and MBT (10), and raise up to 50% for TFP (11). Treatment response of 50% indicate, that still half of the patients do not profit from treatment (12). Borderline personality disorder is prevalent in clinical populations and the cost of treatment is high (13, 14). For these reasons, research on effective shorter term treatments for BPD is warranted, also concerning different indication. One specific facet of BPD may be potentially amenable to intervention. It is increasingly clear that recurring mental images of future episodes of self-harm increase the likelihood of EDB (15). Mental images of suicidal imagery are predictive of suicide attempts (16). Cloos and colleagues (17) conducted an internet survey of 912 clinical and non-clinical adults and found that higher rates of distressing negative images were correlated with EDB. An investigation of 393 university students revealed that when experiencing the urgent need to perform self-injurious behaviors, almost 74% thought about self-harming in the form of mental images (18). Furthermore, a study of 10 self-injuring adults indicated that their first mental image about self-harm amplified the urge to self-injure and occurred directly after the first self-harm attempt (19). Increased levels of mental imagery are associated with suicidal ideation (20) and self-harming behavior (21). In a sample of inpatients and outpatients suffering from BPD, almost 68% reported intrusive mental imagery about EDB (21), and about 91% of the mental images were prospective in nature, regarding future episodes. New therapeutic approaches are needed to address and manage these experiences.

Imagery rescripting (IR) techniques were originally developed to treat trauma-related intrusions in individuals suffering from posttraumatic stress disorder (22). The case for using IR to manage intrusive mental imagery has been strengthened by a recent meta-analysis describing efficacy and large effect sizes in the treatment of aversive memories occurring in the context of numerous mental disorders (23). The techniques have been effectively utilized in the treatment of major depression (24, 25), anxiety disorders such as social phobia (26, 27), specific phobia (28, 29) or test anxiety (30), eating disorders (31), body dysmorphic disorder (32), and nightmares (33).

Traditionally, IR techniques have been employed to fulfill retrospective emotional needs in the treatment of personality disorders. Disturbing mental images are modified into positive and pleasing reconstructions, which in turn facilitates more successful coping in aversive situations (11, 34, 35). For example, a childhood experience of being locked in a closet and verbally abused by a parent can be modified *via* imagery: the other parent enters the room, frees the child from the closet and comforts him or her, creating a new emotional experience of being safe, assisted, and valued by the other parent. As positive prospective mental imagery has already been used in depression to boost optimism (36), we surmised that the application of IR techniques could hold promise in the redirection of negative emotion and future behavior.

With this in mind, we devised a two-session intervention using IR techniques to be utilized in the treatment of BPD. The individual sessions would entail the reconstruction and modification of mental imagery about EDBs. The intervention would require daily rehearsal at home, providing practice of this process. The design was intended to reduce negative emotions and cognitions regarding self-injurious behaviors in the future by replacing them with more adaptive content. We wanted to enable individuals with BPD to begin coping imaginatively in response to perceptions of hopelessness, and wished to provide direct engagement in this new form of emotional regulation. Our exploratory study investigated the acceptance and efficacy of the treatment approach in females diagnosed with BPD and intrusive mental imagery. We hypothesized that we would see an improvement in emotional regulation and a reduction in borderline symptomatology.

## Methods

### Participants

Seven female in- and outpatients at the University Clinic of Psychiatry and Psychotherapy III, in Ulm, Germany, were included in this study. Since up to 70% of patients seeking specialized treatment for BPD are women, only female patients were included (14). Each had received diagnosis of borderline personality disorder and histories of EDB. Clinical assessments were conducted, inclusion and exclusion criteria were applied, and self-reported measurements were completed. Written, informed consent was obtained prior to participation.

Inclusion criteria were female gender, age between 18–50 years, BPD diagnosis, a history of self-injury (e.g., skin cutting, binge-eating, or other impulsive behaviors), and reports of recurrent, intrusive images of EDB.

Exclusion criteria were diagnoses of a schizophrenia spectrum or bipolar disorder or current substance dependency.

**Figure 1:**
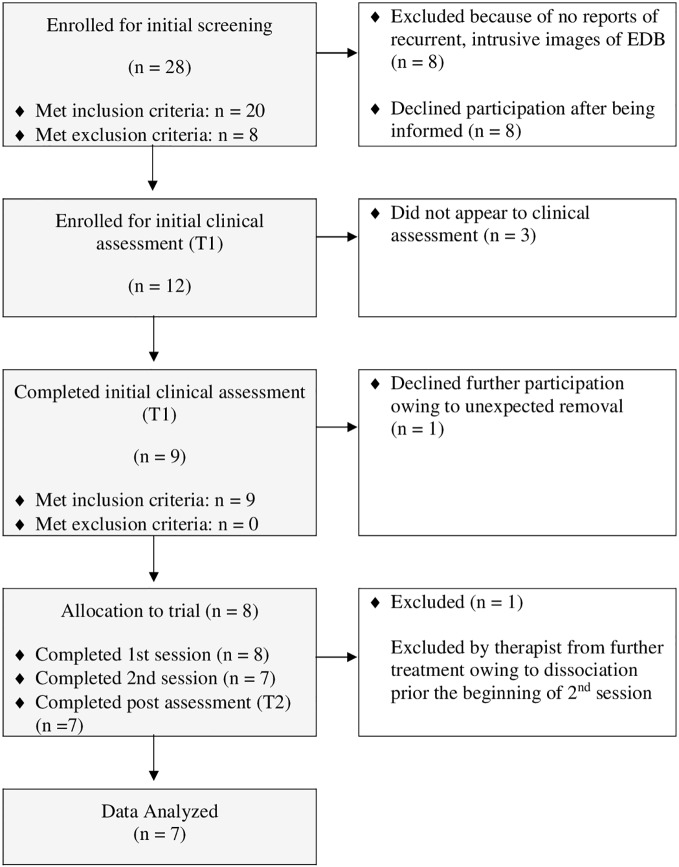
Patient flow chart.

Recruitment began in April 2017 and was finalized in November 2017. In total, 28 individuals were scheduled for interviews and screened. Of these, 20 met inclusion criteria. Out of the 20 eligible participants, 12 declined participation for various reasons: four of these refused because of the distance to the research laboratory; four did not attend the initial assessment and could not be contacted; two did not have sufficient time for the initial clinical assessment; and two chose not to interrupt their current psychotherapy. **Figure 1** shows the CONSORT diagram of participants.

### Intervention

The intervention consisted of two individual IR sessions. During each treatment session, participants were guided to select disturbing mental imagery and to modify it with comfortable rescripting. On average, treatment sessions lasted 95.37 min (*SD* = 22.39).

#### First Session

*(1) Psychoeducation:* The first treatment session began with psychoeducation. The therapist explained the purpose of the study and the concept of mental imagery. To introduce the sensory characteristics of mental imagery, the therapist gave a brief example (e.g., how to give a speech), prior to exploring the participant's mental images about EDB. Later, the metaphor of a “horror movie” was used to explain how negative mental imageries (or a “mental film script”) can be changed into a more benign “storyline” using IR techniques.

*(2) Resurrection of chosen mental imagery:* After the participant chose a vivid and stressful mental image, the therapist instructed the participant to close her eyes, focusing on it in the present from a first person perspective, as vividly as possible. Then she was asked to give a detailed oral narrative regarding the details of what she saw, heard, smelled, tasted, and felt. She was also asked to share her thoughts and feelings about it.

*(3) Identification of point-of-no-return:* The therapist helped the participant isolate a crucial point of no return; that is, a point at which the consequences of performing the EDB would become inevitable. The participant was asked to define thoughts and feelings closely linked to the EDB. Establishing the point-of no return is central to the successful application of this technique. The storyline was changed at this precise moment, facilitating a functional and comfortable fulfilling of emotional needs related to the imagery experience—in lieu of performing the EDB.

*(4) Imagery modification/rescripting:* The therapist then queried the participant for possible events that might diminish the negative emotions, thoughts, and bodily sensations generated by the imagery, and assisted her in creating an alternate ending that fits her emotional needs. Together, the therapist and the participant began the rescripting.

Some participants preferred to cope with the situation on their own, while others preferred the appearance of an additional imagery person. Either way, new idiosyncratic mental imagery was generated, representing the participant's emotional needs. The process ended when the participant was satisfied with the results of the rescripting. For example, one participant initially imagined that she was cutting herself with a craft knife; during the course of imagery rescripting, she replaced taking the craft knife by taking a green Pokémon pen, which reminded her of a good friend, then receiving an unexpected call from her sister. That imagery made her feel beloved and secure.

*(5) Audio tape of modified mental imagery:* To replace the former and to consolidate/integrate the new, the therapist and the participant worked together to produce a written version and an audio tape of the modified imagery, which would be used for imagery rehearsal at home.

#### Homework

The participant was instructed to perform daily rehearsal by listening to her audiotape and thinking about her rescripted imagery. To assess treatment adherence and check the vividness of the mental images with respect to all sensory modalities, the participant was asked to complete an imagery protocol. To check for unexpected side effects, the participant was instructed to fill out the “Diary Card”—a widely utilized approach in the dialectical behavior therapy, assessing dysregulated behavior (37).

#### Second Session

The second treatment session began with the therapist asking how often the participant rehearsed the new imagery during the past week; whether modifications would be necessary in the creation of a second mental rescripting; whether she had experienced any negative effects from the previous treatment session; and whether the participant had any additional questions.

Then, the therapist briefly explained the procedure of IR again and asked the participant to describe another EDB that was accompanied by intrusive mental images. Participants usually reported another form of EDB that was less disturbing than the first. Then, this mental imagery was rescripted following the same procedure practiced in the first session. At the end, the therapist asked the participant about any remaining questions; provided an opportunity for the participant to give feedback on the process; recommended daily rehearsal of the technique; and encouraged the participant to adopt this newly learned technique for any upcoming EDB intrusive images.

For encouragement, the therapist briefly summarized the technique: first, mental imagery about an EDB is mentally resurrected; second, a crucial point of no return is identified; and, last, the participant creates a mental imagery fitting her emotional needs (i.e., makes her feel more comfortable and less distressed).

### Design

To assess the feasibility of this new treatment approach, we implemented a case series with a pre-post design. As it was an exploratory study, there was no control group, and no clinical trial was registered. The study was approved by the ethics committee of Ulm University in Germany.

During a 2-week period, each participant underwent an initial clinical assessment (T1; prior to participation); two individual sessions of IR, administered by an experienced clinical psychologist (first author: CS); and a post-assessment immediately following the second session (T2).

To avoid concurrent treatment effects, participants were scheduled for either an initial clinical assessment (T1) immediately following inpatient treatment (approximately 1 week after discharge from hospital) and prior admission of outpatient treatment, if the patient consented to be contacted; or if participants were already undergoing outpatient psychotherapy, they were asked to take a break of 3 weekly sessions. A post-assessment of self-reported measures was conducted immediately after the second session (T2). To ensure independence and increase objectivity for the non-self-reported measures, rater-blindness was implemented (i.e., a trained clinical psychologist not involved in the treatment conducted the assessments). To ensure adherence to the treatment protocol, the last author (ZSV) administered weekly supervisions for the therapist (CS).

### Measures

#### Diagnostic Interviews

Borderline personality disorder diagnosis was confirmed by a trained and clinically experienced psychologist using the German version of the *Structured Clinical Interview for DSM-IV Disorders* [*SCID-II*; (38)] which is identical to the assessment by the German version of the *Structured Clinical Interview for DSM‐5*-*PD* for assessing personality disorders [*SCID-5-PD*; (39)]. The comorbidity of psychological disorders was assessed *via* the *Mini International Neuropsychiatric Interview* [*M.I.N.I.*; (40)].

The German version of the *Long Form Self-injurious Thoughts and Behaviors Interview* [*SIT BIG;* (41)] was used to assess characteristics of self-injurious behavior and thoughts (EDBs).

During T1, mental imagery of EDBs was assessed *via* an adapted version of the semi-structured *Imagery Interview* (42) and the *Intrusion interview* (43). The original items and questions were used but with EDBs substituted for trauma-related images. In our adapted version, the interviewer first provided participants with a short introduction about the nature of mental imagery; then, to confirm understanding, participants were asked to create an imaginary character and the interviewer explored participants' sensations (sight, hearing, smell, touch and taste), thoughts, and feelings toward the mental imagery of the EDBs.

#### Self-Reported Outcome Measures

The *Borderline Symptom List-95* [*BSL-95*; (44)] was used to measure distress relating to seven different subscales: self-perception, affect regulation, self-destruction, dysphoria, loneliness, intrusions, and hostility. Each of the 95 items was rated on a 5-point Likert scale ranging from 0 (*not at all*) to 4 (*very strong*). There was good internal consistency [Cronbach's α = 0.97; (44)].

To measure emotion regulation processes and differentiate between suppression and reappraisal, the German version of the *Emotion Regulation Questionnaire* [*ERQ;* (45)] was employed. The 10 items were rated on a 7-point Likert scale ranging from 1 (*strongly disagree*) to 7 (*strongly agree*). There was good internal consistency with Cronbach's α above 0.74 for both subscales (45).

The German Questionnaire of Emotion Regulation for Adults (*Fragebogen zur Erhebung der Emotionsregulation bei Erwachsenen* [*FEEL-E*; (46)] was used to measure 12 different emotion regulation strategies, six adaptive and six maladaptive. The *FEEL-E* has 72 items, which are rated on a 5-point Likert scale ranging from 1 (*almost never*) to 5 (*almost always*). Ratings are separately measured for each emotion: anger, fear and sadness [Cronbach's α = .91 for adaptive strategies and .88 for maladaptive emotions regulation strategies; (46)].

The German version of the *Beck Depression Inventory-II* [*BDI-II*; (47)] was used to assess negative mood effects and depressive symptoms. This version has 21 items which are rated on a 4-point Likert scale and have a Cronbach's α of .84 (48).

To assess treatment acceptance, we used three subscales (perceived change, participants' experienced confidence and mental calming down) of the German *Bielefeld Client Experience Questionnaire [Bielefelder Klienten-Erfahrungsbogen;* *BIKEB*; (49)], which assesses the post-session outcomes of a previously provided psychotherapy session to predict treatment efficacy. It is rated on a 6-point Likert scale ranging from 0 (*not at all)* to 5 *(totally agree).* The scale has a Cronbach's α ranging from .74 and .87 for the subscales in this study (49).

### Statistical Analysis

Data were analyzed using IBM Statistics 25 (50) and STATISTICA 13.1 (Dell Incorporation, 2016). Wilcoxon test was conducted to measure overall changes over time. As this was an exploratory study, we did not apply a Bonferroni adjustment for multiple comparisons. Calculation of Cohen's *d* for repeated measures was computed as described by Morris & DeShon (51). Values of .20 were interpreted as weak, .50 as medium, and .80 were interpreted as representing large effects (52). The alpha error was set at 0.05, but because of the preliminary character of our study, *p*-values up to 0.1 were interpreted as trends.

## Results

### Sample Characteristics

In total, eight females with BPD were enrolled in this study, but only seven had their data analyzed and the latter were classified as completers because they participated in both treatment sessions and all assessments. Participants were aged 20–35 years (*M* = 24.86, *SD* = 5.93) and met an average of 6.71 out of nine diagnostic criteria for BPD (*SD* = 1.38). On average, participants reported performing 7.43 different types of EDBs (*SD* = 2.07), the most frequent being: NSSI (skin cutting: *n* = 7; 100%), binge-eating (*n* = 6; 86%) and impulsive behavior (*n* = 5; 71%). All participants had comorbid diagnoses, and averaged 3.14 different diagnoses (*SD* = 2.34). Additionally, 71% (*n* = 5) reported suicidal ideation. All participants were receiving stable medication.

During the first treatment session, six participants mental imagery about skin cutting as a proxy of EDB and one chose imagery about binge-eating; during the second: four chose mental imagery about NSSI (one reported hitting, one cutting, one scratching, one burning), two chose imagery about binge-eating, and one chose risky driving. See **Table 1** for additional details.

**Table 1:** Patients' mental imagery regarding emotional dysregulation and respective imagery rescripting.

ID	Session 1	Imagery Rescripting	Session 2	Imagery Rescripting
1	Skin-cutting with a cutter	Phone call from her sister	Hitting fingers with a door	Taking a shower and selfcare
2	Skin-cutting with a razor	Phone call from a friend	Skin-cutting with a razor	Spending time with a friend
3	Skin-cutting with a razor	Spending time with her dog	Disturbing wound-healing	Taking care of pets
4	Skin-cutting with a razor	Spending time with her children	Binge-eating	Picture and memories of a good friend
5	Skin-cutting with a knife and drinking wine	Spending time with her partner	Risky driving	Spending time with her partner
6	Binge-eating	Preparing a healthy meal	Binge-eating	Spending time with a friend
7	Skin-cutting with a knife	Spending time with a friend	Burning with a cigarette	Phone call from her sister

### Treatment Acceptance and Satisfaction

All participants allocated to the treatment appeared for both treatment sessions. One participant was excluded from further participation at the beginning of the second session because of dissociative symptoms. However, this patient appeared with dissociative symptoms to the waiting room of the laboratory, making the application of grounding techniques necessary. A further exploration revealed that the dissociative symptoms were triggered by an interpersonal conflict prior appearance to the second session, thus making an relation to the intervention very unlikely. Participants reported that they practiced their daily homework on average 4.2 days per week (*SD* = 2.17). Six participants reported the treatment as “helpful” immediately after participation; six reported that they would recommend the treatment; four reported that they felt better after the rehearsal of imagery; three reported a reduction of EDBs after participation; no participant reported the treatment as “not helpful” although two reported that they worried about being triggered through imagery rehearsal when highly distressed. Three reported that it was difficult for them to perform daily rehearsal, especially at the weekends. According to *BIKEB* results, global satisfaction with treatment sessions was good *(M* = 4.57*, SD* = 0.79). The mean score of perceived change was 3.64 (*SD* = 0.97). The mean score of experienced confidence was 3.04 (*SD* = 1.20), and the mean score of mental calming was 3.75 (*SD* = 0.50).

### Efficacy

**Table 2** shows the mean total scores and standard deviations for the *BSL-95, ERQ, FEEL-E,* and *BDI-II* for all participants at T1 and at T2.

**Table 2:** Mean total scores and standard deviations for the BSL-95, ERQ, FEEL-E, and BDI-II for all participants at T1 and at T2.

	Pre *M (SD)*	Post *M (SD)*	Pre-Post Effect size^a^
**BSL-95**			
Total	184.29 (76.61)	151.29 (94.66)	.83
**ERQ**			
Cognitive Reappraisal	2.88 (1.34)	3.07 (1.76)	.14
Expressive Suppression	4.29 (1.60)	3.96 (1.58)	.33
**FEEL-E**			
Adaptive Strategies	85.71 (28.13)	91.86 (30.81)	.41
Maladaptive Strategies	132.14 (19.18)	124.14 (24.90)	.81
**BDI-II**			
Total	32.57 (14.52)	29.14 (16.04)	.70

BSL-95, Borderline Symptom List-95; ERQ, Emotion Regulation Questionnaire; FEEL-E, Questionnaire for Emotion Regulation for Adults; BDI-II, Becks Depression Inventory.

a Cohen's d for repeated measures.

#### Severity and EDB

With respect to borderline symptoms, no significant symptom exacerbation was detected with respect to the *BSL-95* total scores. Wilcoxon test showed a trend toward symptom reduction from T1 to T2 (*z* = -1.690, *p* =.055).

#### Emotion Regulation

With respect to adaptive emotion regulation strategies, we found no statistically significant deterioration over time (*z* = -.847, *p* =.227). Generally, maladaptive strategies showed a trend toward improvement over time (*z* = -1.521, *p* =.078). Regarding emotion regulation strategies of anger: maladaptive strategies showed a trend for reduction from T1 to T2 (*z* = -1.682, *p* =.063) but no significant difference was observed for the adaptive strategies (*z* = -1.051, *p* =.172). No significant differences over time were observed for adaptive or maladaptive emotion regulation strategies, when comparing the emotions: fear (adaptive: *z* = -.339, *p* =.398; maladaptive: *z* = -.106, *p* =.500) and sadness (adaptive: *z* = -1.335, *p* =.125; maladaptive: *z* = -.841, *p* =.234).

No deterioration over time was observed according to *ERQ:* neither cognitive reappraisal (*z* = -.105, *p* =.500), nor expressive suppression did significantly change over time (*z* = -.530, *p* =.359).

#### Depressive Symptoms

No symptom exacerbation with respect to depressive symptoms was detected. Instead, we found a trend for decrease from T1 to T2 (*z* = -1.472, *p* =.094).

## Discussion

This study presented a tailored, short intervention comprising two individual sessions of IR and the rehearsal of rescripted mental imagery related to EDB (e.g., skin cutting, binge eating, and impulsive behavior) in participants suffering from BPD. Acceptance of the new short intervention was good: All participants in the study attended both treatment sessions, except for one woman. This patient did not receive the second treatment because she experienced dissociative symptoms prior the beginning of session 2 as a result of an preceding interpersonal conflict so that only grounding and reorientation techniques were applied by the therapist. No participant dropped out during the post assessment. Our findings are corresponding to other feasibility studies using short-term interventions of IR to reduce symptom severity, also reporting no dropouts (53–55). All participants reported daily imagery rehearsal of the new, rescripted storyline, indicating 100% participant adherence. All participants expressed that the treatment was helpful and that they would recommend it. Global satisfaction with the treatment was very good, and all participants perceived subjective change, mental calming, and the development of confidence after the treatment sessions, according to the results from the *BIKEB*. Further, their reports were in line with clinical observations by the therapist. Moreover, most participants appeared to have higher levels of arousal prior compared to post application of IR.

We did not observe a deterioration with regard to borderline symptoms, and severity declined over time. Particularly, auto-aggression in terms of self-harm and loneliness showed a trend toward reduction. Although auto-aggression directly relates to the EDB construct, which was targeted by our intervention, loneliness could reflect a more indirect proxy of EDB. Loneliness is reported to precede self-harming behaviors (21, 56), which in turn are carried out for the sake of emotion regulation. Although our results do not allow for similar causal inference, we still cannot rule out that successful application of IR may not only improve functional emotion regulation strategies (making EDB less frequent) but also enhance positive affect (leading to aversive feeling reduction, such as loneliness). Consistent with this hypothesis, we found that maladaptive emotion regulation strategies (results from *FEEL-E*) tended to decline. Higher scores on this subscale indicate the performance of dysfunctional behaviors such as social withdrawal. During IR, participants developed new story lines, including seeking help through social interaction (e.g., calling a friend instead of cutting one's self). Thus, imagery of positive helpful social interactions may have led to less social withdrawal in real life, as imagery of future behavior has been shown to increase the likelihood of that specific behavior happening (15, 57).

In contrast, adaptive emotional regulation strategies (also results from *FEEL-E*) did not increase. Higher scores on this subscale denote the performance of functional behaviors such as problem solving or acceptance of personal emotions; however, during IR, no participant developed story lines with elements of classical problem solving, and negative affect was changed, not accepted. Shifting bad thoughts to more soothing ones was consistent with nearly all new story lines we saw. These findings are consistent with the central premise of IR techniques: that is, the fulfilling of emotional needs by the generation of positive and pleasing imagery instead of cognitive problem solving (35).

Emotion suppression did not improve over time. Higher scores in this subscale suggest hiding one's feelings during social interactions. Nonetheless, improving such behaviors was not the intent of our IR intervention. Moreover, participants regularly included helpful social interactions and asked for help in rescripted imagery. As indicated by lower *BDI-II* scores, changes in negative affect were observed over time.

In summary, we found moderate to high effect sizes regarding changes in borderline symptom severity and emotion regulation strategies. These findings are comparable to previous studies using a maximum of three sessions of IR techniques to reduce symptom severity in other psychiatric disorders (23, 27, 53).

The observed changes in emotion regulation and rates of EDB can be explained by mechanisms of mental imagery: First, the mental imagery of crucial situations elicits the same neural activations as directly perceived, real experiences (58). Second, the imaging of future acts influences future behavior (59). New mental representations related to new behaviors and coping with emotional distress, rather than self-harm, may enhance real coping in future situations. Further, every participant generated mental imagery, which directly satisfied their individual emotional needs and led to less perceived distress. Daily practice of the modified mental imagery may have strengthened this affective aspect of the newly created neural pathway.

In conclusion, our findings indicated that our new IR short intervention—and its rehearsal—might facilitate the management of negative emotions and perceptions of hopelessness by the creation of a new coping method. The potential benefit of the intervention was also demonstrated by the absence of symptom exacerbation.

## Limitations

Generalizability of the present findings is limited because of the small sample size, lack of a control group, and because the sample contained solely female participants.

Moreover, it remains unclear if treatment effects and symptom improvement only apply to the two-session short intervention. Non-specific treatment effects (e.g., therapeutic alliance) cannot be excluded, but are considered unlikely due to the very brief window (i.e., 2 weeks). Therefore, replication in randomized controlled trials should be conducted to confirm the potential efficacy of the intervention. Optimal results will be obtained with a larger sample, a control group, a follow-up assessment of long-term effects, and an assessment of confounding effects.

## Conclusion

Our findings indicated acceptance and potential efficacy of this new short intervention which consists of two stand-alone IR sessions. To our knowledge, this was the first study to modify mental imagery concerning future EDB in participants suffering from BPD. In the event of future validation in controlled studies, the proposed program might be a promising treatment component in the amelioration of emotion dysregulation for individuals with BPD, and could be integrated into more comprehensive behavioral approaches, such as dialectal behavior therapy.

## Data Availability Statement

The datasets generated for this study are available on request to the corresponding author.

## Ethics Statement

The studies involving human participants were reviewed and approved by Ethical Board of the University of Ulm, Germany. The patients/participants provided their written informed consent to participate in this study.

## Author Contributions

CS: Study conception and design, analysis and interpretation of data, manuscript writing and editing, treatment conduction. JK: Study design and manuscript editing, AM: Study design and manuscript editing, BC: Study design and manuscript editing, ZS-V: Study conception and design, interpretation of data, manuscript editing, critical revision, treatment supervision.

## Conflict of Interest

The authors declare that the research was conducted in the absence of any commercial or financial relationships that could be construed as a potential conflict of interest.
